# Pollinivory and the diversification dynamics of bees

**DOI:** 10.1098/rsbl.2018.0530

**Published:** 2018-11-14

**Authors:** Elizabeth A. Murray, Silas Bossert, Bryan N. Danforth

**Affiliations:** Department of Entomology, Cornell University, Ithaca, NY 14853, USA

**Keywords:** bees, Apoidea, pollinivory, oligolecty, diversification

## Abstract

Pollinivory—the consumption of pollen rather than arthropod prey—is a defining feature of bees (Anthophila; the flower lovers). In virtually all bee species, larvae consume a diet composed of pollen mixed with nectar or floral oils. Bees arose from within a group of solitary, carnivorous, apoid wasps in the Early to Mid-Cretaceous, coincident with the rapid rise of flowering plants. It is assumed that the switch from carnivory to pollen-feeding was a key innovation that led to the rapid diversification of bees, but this has never been examined empirically. Here, we explore the hypothesis that pollinivory led to the increased diversification of bees. In contrast to common perception, we find that the switch to pollen-feeding *per se* does not explain their extensive diversification. Rather, our results indicate that pollinivory was a necessary but not sufficient condition for diversification, and that other complementary innovations, such as a broadening of host-plant diet, allowed the diversification of the major bee lineages. Our results have broad implications for understanding tempo and mode of bee diversification dynamics in light of their floral resources.

## 1. Introduction

**Figure 1.**
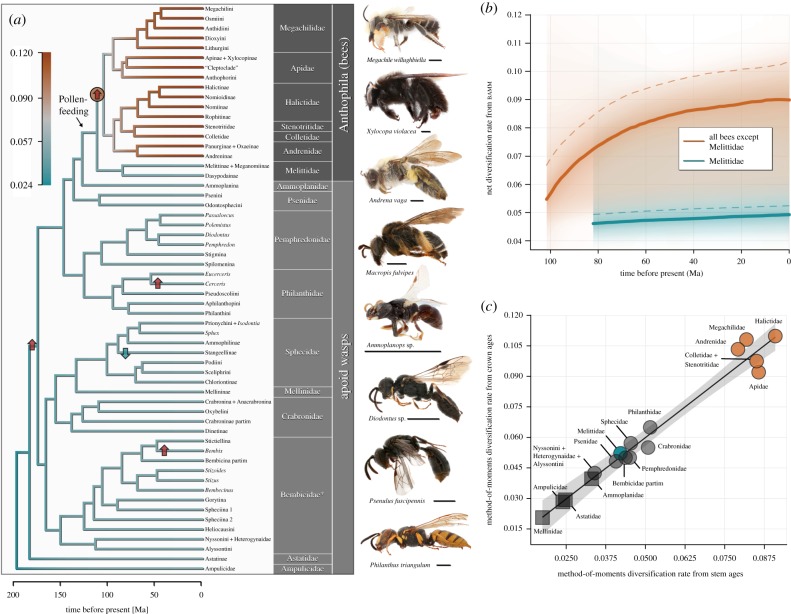
Diversification dynamics of bees (Anthophila) and apoid wasps. (*a*) Dated phylogeny of Apoidea, based on Sann *et al.* [2]. Branches are coloured according to net diversification rates from bamm. The rate configuration with the highest posterior probability consisted of a single rate shift (*f* = 0.67), which is indicated by the red circle. The arrows indicate increases (red) and a decrease (blue) in diversification rates (*r*) identified through the maximum-likelihood-based method medusa (electronic supplementary material, figures S1–S4). We found no significant support for an increase in diversification along the branch of bees on which the evolutionary transition to pollen-feeding occurred. Family names follow the new classification of Sann *et al.* [2], and the chronogram corresponds to their tree 3. The family Bembicidae is followed by an asterisk owing to the placement of another family (Heterogynaidae) within. Scale bars next to specimens correspond to 2 mm. (*b*) Profiles of the two different diversification dynamics within bees, as identified by bamm. The average net diversification rate (solid lines) is 0.051 for Melittidae, and 0.080 for the remaining bee lineages combined. Dashed lines show speciation rates. (*c*) Clade-specific diversification rates from the phylogeny-independent method-of-moments estimator with *ɛ* = 0.5. Net diversification is inferred from both stem and crown ages. The shaded area indicates the 95% CI of the linear regression (*F*_1,11_ = 221.4, *p* ≤ 0.0001, *R*^2^ = 0.948, from the 13 samples for which stem and crown ages are available). Four groups did not have crown ages and are represented by square symbols. These are plotted at their stem-based rate value, but are placed directly on the regression line as an estimate of their crown-based rates. Lineages that mainly comprise generalist pollen-feeding species (orange) have higher mean diversification rates than the remaining groups (two-sample *t*_14.99_ = 12.452, *p* < 0.0001; based on stem age values).

With more than 20 000 described species, bees are a successful, speciose and widely distributed lineage of angiosperm pollinators [1]. In virtually all bee species, larvae consume a diet composed of pollen mixed with nectar or floral oils. Bees arose from within a group of solitary, carnivorous, apoid wasps in the Mid-Cretaceous (figure 1), coincident with the rapid rise of flowering plants [3]. In order to accurately determine the impact of pollinivory on bee diversification, one needs to correctly infer the sister group to the bees. Previous studies based on both morphological and molecular data have identified a variety of potential bee sister groups, including all apoid wasps, the family Crabronidae and the crabronid subfamilies Philanthinae and Pemphredoninae [4]. A recent study offers a new perspective on the origin of bees. Sann *et al.* [2] used a massive phylogenomic dataset including 93 species of apoid wasps and 43 species of bees to reconstruct the phylogeny of Apoidea, and identified the small-bodied, thrips-hunting Ammoplanina as the extant sister group to the bees.

Classic and contemporary studies have shown that the switch to a herbivorous diet increased diversification of major insect lineages [5,6]. From an evolutionary perspective, plants represented a new ecological niche, providing both abundant and diverse food resources. Comparably, the transition from carnivory to the specialized herbivorous lifestyle of pollinivory is assumed to be a key factor that triggered the diversification of bees [7,8]. While this view seems plausible, it has never been empirically tested. To answer this question, we used the most comprehensive apoid phylogeny to date [2], in combination with current data on species richness for all major clades (electronic supplementary material, table S1).

## 2. Material and methods

We used the dated phylogeny of Sann *et al.* [2], which has a crown age of bees intermediate between the two most recent phylogenomic studies [7,8], and pruned it to accurately represent species richness estimates for bees and apoid wasps (see the electronic supplementary material). We calculated diversification rates (defined as the rate of speciation minus extinction) using three different methods. Two are tree-based approaches that require a dated phylogeny and sampling fractions to account for incomplete taxon representation, and our third method is based on extant species richness and clade age. First, we used bamm (Bayesian Analysis of Macroevolutionary Mixtures) [9] to identify rate shift configurations on the phylogeny, sampling from a posterior distribution of possible macroevolutionary scenarios. Second, we used medusa (Modelling Evolutionary Diversification Using Stepwise AIC) [10] to calculate the best-fitting diversification scheme by progressively optimizing rate shifts on the tree. However, both bamm and medusa have been criticized for theoretical and statistical shortcomings [11–13], and there are conflicting views on the use of these programs for empirical data [14].

In order to corroborate diversification rate estimates of bamm and medusa, we lastly used method-of-moments estimators [15], where diversification rates are calculated individually on a per-clade basis. This method requires values for species richness and clade age, and allowed us to incorporate different estimates of the relative extinction fraction (*ɛ* = 0, 0.1, 0.5, 0.9). We calculated the net diversification rate of a clade under both the crown age (the node of the most recent common ancestor of all extant clade members) and the stem age (the node ancestral to the crown group node). A detailed version of all methods, including the diversification analyses for all four estimated chronograms of Sann *et al.* [2], can be found in the electronic supplementary material.

## 3. Results and discussion

Surprisingly, none of our analyses showed that Anthophila as a whole diversified faster than their wasp relatives (figure 1*a*; electronic supplementary material, figures S1–S5; tables S2 and S3). Contrary to the prevailing pattern of herbivory-linked diversification increases across insects [5,6], the shift to a pollen-based diet within the Apoidea was not accompanied by increased diversification rates. Specifically, we found no detectable rate shift along the basal branch of bees—the branch along which the evolutionary transition to pollen-feeding must have occurred. This means that pollinivory, the characteristic feature separating bees and related wasps, cannot explain the enormous diversity of bees that we see today. Instead, both tree-based approaches independently identified a significant diversification increase within Anthophila, along the branch leading to all bees *excluding* the family Melittidae. The medusa analyses unambiguously identified this rate shift on all four alternative trees (electronic supplementary material, table S2). bamm inferred a rate shift along this branch on all input trees, and for two it is the most probable rate shift regime (*f* = 0.54, *f* = 0.67, figure 1*a*; electronic supplementary material, figures S1 and S3). In this scenario, the bees excluding Melittidae have a relative extinction fraction that is more than twice as high as that of melittids and other apoid groups (*ɛ* = 0.17 versus *ɛ* ≤ 0.08), yet is offset by a much higher speciation rate (0.097), which results in an increased rate of net diversification (electronic supplementary material, table S3). This shows that Melittidae are not species-poor just because of greater extinction. However, bamm results from two trees show it is also plausible that there is no significant rate shift across the phylogeny (electronic supplementary material, figures S2 and S4).

Evidence for a shift within the bees that is not coincident with the origin of pollinivory is congruent with our method-of-moments estimates of diversification rates (figure 1*c*). Melittidae diversified slowly, with a stem age-based rate (0.042 with *ɛ* = 0.5) just slightly higher than that of the closest wasp relatives of bees (Ammoplanidae; 0.033), and lower than several other groups of apoid wasps, such as Philanthidae (0.051), Sphecidae (0.045) and Crabronidae (0.051). This pattern holds true under all four tested relative extinction fractions (electronic supplementary material, figure S5). Both Melittidae (203 described species) and Ammoplanidae (123 described species) are relatively species-poor groups of Apoidea. By contrast, the remaining bee lineages comprising over 20 000 described species diversified much more rapidly (*r* = 0.081 with *ɛ* = 0.5) than Melittidae.

The two likelihood-based methods, bamm and medusa, should arguably be used with caution on empirical datasets, as suggested by recent evaluations of the programs [11–13]. Further, bamm is less suited for diversification analyses on phylogenies with very incomplete taxon sampling, such as the one used here, and our sampling regime precludes the detection of shifts below the subfamily level. Nonetheless, the diversification rates of apoid families inferred by both the method-of-moments estimators and bamm are in fact very similar (electronic supplementary material, table S2), and all methods converged on a scenario in which the diversification rate of Melittidae is much lower than for the remaining major bee clades.

What key biological features distinguish Melittidae from other bees? Melittids are a small enigmatic family of strictly solitary, ground-nesting bees, with a widespread biogeographic distribution. They are almost exclusively narrow host-plant specialists (oligoleges), some with morphological and/or physiological adaptations to efficiently handle specific floral resources [1,16]. For example, certain oil-collecting Melittinae have extremely long forelegs to access deep flower spurs [17], or are able to perceive specific chemical cues to locate floral hosts [18]. The common ancestor of all bees was most probably oligolectic [19], and subsequent transitions to a broad pollen diet required overcoming physiological and neurological constraints [20]. The broadening of host-plant preferences in lineages other than Melittidae may have been one factor that allowed an increased diversification in the non-melittid bees. The narrow host-plant preferences of the majority of melittid bees putatively put limits on their diversification relative to other bees.

We provide new insights into diversification in bees and related wasps. Our study opposes the conventional thought that bees diversified due to the evolutionary novelty of pollinivory, but suggests that pollen-feeding may be a necessary though not sufficient condition for diversification. With a broadening of host-plant preferences, bees may have been able to ‘escape from oligolecty’ and become the dominant flower-loving, pollinivorous lineage on the Earth.

## Supplementary Material

All supplementary material.

## Data accessibility

Figures S1–S5 and tables S1–S3 can be found in the electronic supplementary material.
