# Supplementary material for: Pollinivory and the diversification dynamics of bees
Source: Biol Lett. 2018 Nov 14;14(11):20180530. doi: 10.1098/rsbl.2018.0530 (PMC6283915; doi:10.1098/rsbl.2018.0530)
Supplement: All supplementary material. [file rsbl20180530supp1.pdf]

Electronic Supplementary Material

Pollinivory and the diversification dynamics of bees

Elizabeth A. Murray[†,‡], Silas Bossert[†,‡], Bryan N. Danforth

Department of Entomology, Cornell University, Ithaca, NY 14853, USA

[†] These authors contributed equally
[‡] current address: Department of Entomology, National Museum of Natural History, Smithsonian Institution, Washington, DC, 20560, USA

ORCID iD: EAM 0000-0001-9300-3419; SB 0000-0002-3620-5468; BND 0000-0002-6495-428X

Supplementary material and methods

Dated phylogeny and species richness estimates

The dated phylogenies of Sann *et al.* [1] (their figure 2 and supplementary trees 1-4) were pruned to include just Apoidea – this includes all of the bees plus the carnivorous wasp families Heterogynaidae, Ampulicidae, Sphecidae, and Crabronidae, which is not a monophyletic family (following the classification of Pulawski [2]). For the diversification rate analyses, we obtained species counts across the major clades of Apoidea. We included the numbers of presently described species for the major lineages of bees from the discoverlife.org database [3] and obtained species numbers for the apoid wasps from the online Catalog of Sphecidae *sensu lato* [2].

As species richness is generally not equally distributed among clades, we collapsed nodes at which the taxon sampling did not allow precise assignments of species numbers. This way we avoided potentially misleading sample fractions, while minimizing the loss of topological information of the phylogeny. For example, we retained the four clades of Halictidae that represent the four subfamilies, and coded each clade with the respective number of described species for each subfamily. However, we collapsed each subfamily into a single tip, as the taxon representation of Sann *et al.* [1] did not allow exact assignment of species numbers for shallower divergences (i.e., tribes). In summary, our final phylogeny had 58 tips. Each phylogeny had the same topology, but with different node age estimates. The root ages of each pruned tree in millions of years (Ma) were: tree 1 = 194.58 Ma (figure S1), tree 2 = 181.54 Ma (figure S2), tree 3 = 193.14 Ma (figures 1A, S3), and tree 4 = 185.78 Ma (figure S4).

In order to ensure unbiased coding of monophyletic groups, we adjusted the estimation of sample fractions as follows. The subfamily Apinae (Apidae) is paraphyletic in respect to Xylocopinae, Anthophorini, and all cleptoparasitic Apinae except *Ctenoplectrina* and the parasitic Euglossini [4-6]. We therefore combined the numbers of the described species for Xylocopinae plus Apinae, and subtracted the species number of the cleptoparasitic Apinae (except for *Ctenoplectrina*, *Aglae* and *Exaerete*) and Anthophorini. We then estimated separate sample fractions for the tips representing the Anthophorini and the 'cleptoclade' sensu Cardinal *et al.* [5], and pruned the chronogram accordingly. This ensures unbiased coding and that the species numbers of all apid clades sum to the number of all described species (5840 species). We estimated sample fractions for all tribes of Megachilidae, except for the species-poor subfamily Fideliinae (22 species), which were not sampled in the initial data set. However, the calculated

sample fractions ensure the representation of 99.46% of megachilid taxa. The sample fractions of Halictidae were coded on subfamily level, and Stenotritidae are represented by the single species *Ctenocolletes rufescens*. As only 2 of 5 subfamilies of Colletidae are present in the data set, we collapsed the clade of Colletidae into a single terminal that represents the entire family. The Andrenidae were coded on subfamily level except for Alocandreninae and Oxaeinae. The single species of Alocandreninae (*Alocandrena porteri*), was added to the 1552 species of Andreninae, and the 22 species of Oxaeinae were combined with the described number of species for Panurginae, as these subfamilies were inferred to be sister taxa [7]. Lastly, we coded the Melittidae on subfamily level except for Meganomiinae, which are not present in the phylogeny. As Meganomiinae are sister to Melittinae [7], we assigned the number of described species of both subfamilies to the terminal of the Melittinae.

We estimated sample fractions for the apoid wasps based on the phylogenetic results of Sann *et al.* [1], but followed the classification of Pulawski [2] to specify the clades for which we assigned the sample fractions (table S1). We collapsed the following clades into one terminal each and assigned species numbers for the respective groups: Psenini, Stigmina, Pseudoscoliini, Aphilanthopini, Philanthini, Gorytina, Stictiellina, Heliocausini, Alyssontini, Oxybelini, Ammophilinae, Chloriontinae, Podiini, Sceliphrini, Stangeellinae, Astatinae, and Ampulicidae.

Three tribes or subtribes had complete genus representation, which allowed us to code these clades with the species numbers for each genus: Pemphredonina (*Diodontus*, *Passaloecus*, *Pemphredon*, *Polemistus*), Cercerini (*Cerceris*, *Eucerceris*), and Stizina (*Stizus*, *Stizoides*, *Bembecinus*). The following tribes, subtribes or subfamilies were represented only by a single individual sample and were coded accordingly: Odontosphecini (5 spp.), Spilomina (194 spp.), Dinetinae (12 spp.), Mellininae (19 spp.), and Stangeellinae (1 sp.).

Due to paraphyletic groupings of certain suprageneric taxa, we needed to adjust the species representation estimates to ensure unbiased representation of monophyletic groups:

1. The species-rich tribe Miscophini (574 spp., Crabroninae) is paraphyletic. We therefore needed to collapse the crabronine clade which includes the tribes Gastrosericina, Larrina, Miscophini, Trypoxylini, and Palarini into a single branch in order to avoid misrepresentation of the two clades of Miscophini. This terminal is referred to as Crabroninae partim (2787 spp.).

2. The subtribe Spheciina is paraphyletic and required the estimation of two separate sample fractions. We coded Spheciina_1 with the species number of *Sphecius* (= 21 spp.), and Spheciina_2 with the combined estimate for *Tanyoprymnus* and *Ammatomus* (= 31 spp.). As the spheciine genus *Kohlia* was not represented, it was not possible to account for its three described taxa. We further ignored the ‘misplaced’ *Ammatomus* sp. I (Spheciina) from the chronogram [1] and assumed that the specimen was misidentified, as the original article does not provide an explanation of this unlikely placement.

3. The placement of *Anacrabro* renders the subtribe Anacrabronina paraphyletic. We therefore needed to combine the subtribes Anacrabronina and Crabronina to retain a monophyletic group, and collapsed it into one terminal (1498 spp.).

4. The species-rich genus *Bembix* (330 spp.) renders the subtribe Bembicina paraphyletic. We therefore estimated the sample fraction of Bembicina without the 330 species of *Bembix* and coded this genus separately. This terminal is referred to as Bembicina partim (108 spp.).

5. The placement of *Isodontia*, which is one of the two genera of Sphecini, renders the tribe Sphecini paraphyletic. We therefore subtracted the species number of *Isodontia* from the total number of species for the tribe Sphecini. We then combined the species number with the tribe Prionychini (79 spp.) to ensure a coding of the clade of Prionychini + *Isodontia* (total of 141 spp.).

6. The rogue taxon Heterogynaidae was inferred to be nested within Nyssonini (Bembicidae). We therefore combined the species numbers for these groups (236 spp.).

As the phylogeny of Sann *et al.* [1] did not include samples of Entomosericini (3 spp.), Bothynostethini (18 spp.), Eremiaspheciinae (17 spp.), Exeirina (113 spp.) and Handlirschiina (5 spp.), we were not able to account for the diversification of these lineages. However, the total number of excluded species is 159 (the above-mentioned tribes + the genus *Kohlia*) out of 9881 described apoid wasps, yielding a total representation of 98.39%.

The exact species numbers and the calculated BAMM sample fractions are listed in table S1.

Diversification rate analyses

1) BAMM
Rate shift regimes on each of the four pruned phylogenies from the Sann *et al.* [1] supplementary information were inferred in a Bayesian framework using BAMM v2.5.0 [8]. BAMM (Bayesian Analysis of Macroevolutionary Mixtures) models speciation and extinction rates and identifies rate shift configurations on the dated phylogeny. The program uses rjMCMC (reversible jump Markov Chain Monte Carlo) to explore the possible macroevolutionary regimes. This analytical procedure involves calculating the likelihood of a phylogeny under various rate parameters (speciation, extinction, and time-dependent effects of branch age) in conjunction with identifying the location of transitions. Shifts can occur anywhere on the tree (at nodes or along branches), and rates are heterogeneous through time and across lineages.

We ran BAMM for 500 million generations, sampling every 50,000 generations. We used the 'setBAMMpriors' function in the R package BAMMtools v2.1.6 [9] to designate priors appropriate for the tree size. The Poisson rate prior was set to 1.0, which is suggested for trees with fewer than 500 tips. The sampling fraction was designated according to table S1, with backbone sampling set to 0.98. This value corresponds to the total sampling fraction of the apoid wasps, in order to account for the potential of unsampled branches. Following the BAMM analysis, the R package coda v0.19-1 [10] was used to assess MCMC chain stationarity. We discarded the first 10% as a burnin, yielding an ESS >840 in all four trees for both the number of shifts and the log likelihood. BAMMtools was used for subsequent calculations and data visualization. Lastly, we calculated mean net diversification rates using the speciation and extinction values output from the function 'getCladeRates' (reported in table S2).

2) MEDUSA
We used the function 'medusa' in the R package geiger v 2.0.6 [11] as a maximum likelihood-based corroboration of the Bayesian results. MEDUSA (Modeling Evolutionary Diversification Using Stepwise AIC) [12] calculates diversification rate shifts by fitting alternative models on the dated phylogeny. From an initial model specifying one background rate across the phylogeny, rate change breakpoints are inserted successively. Diversification shifts, i.e., significant decreases or increases from the background rate, are calculated under mixed Yule and birth-death models, with shifts occurring at nodes. We designated the species richness represented by each tip as shown in table S1.

3) Method-of-moments estimator

The method-of-moments estimator approach is a theoretically distinct method for estimating clade net diversification. The major difference between this method and the previous two is that the method-of-moments is not tree-based. BAMM and MEDUSA rely on a dated phylogeny and calculate diversification rate shifts in the context of the whole phylogenetic tree. The method-of-moments estimator is different in that it does not furnish rate shifts on a phylogeny. Net diversification rates are calculated on a clade-by-clade basis, using input values of present day species richness and the stem or crown age. This yields the speciation rate, so therefore the usage of a clade-wide relative extinction fraction (epsilon, ε, the ratio of extinction to speciation) has been recommended [13,14]. This calculation ultimately produces a net diversification rate value (speciation minus extinction). In figure 1c, we display results from inputting an epsilon of 0.5 for both the crown and stem age rate estimates. This has been used as an intermediate value in a previous study [15] and has been found in a simulation-based study to be an appropriate value for stem age-based rates [14]. Figure S5 shows diversification results when using the alternative epsilon values 0, 0.1, 0.5, and 0.9.

For the method-of-moments estimator, we calculated bee diversification rates on a family-level basis, and calculated rates for monophyletic groups of apoid wasps as shown in table S2. Four groups of apoid wasps did not have crown ages, therefore these stem values are represented as square symbols and were drawn on the regression line subsequent to its calculation with clades having complete data. Additionally, we conducted a Welch two-sample *t*-test – dividing the values into two groups by their stem age-based diversification rates (when using an epsilon of 0.5): "high" rate values >0.079 (all bee families except Melittidae), and "low" rate values <0.052 (Melittidae and the apoid groups).

Supplementary tables and figures follow:

Table S1: Taxa included in our analysis, based on the apoid phylogeny of Sann *et al.* [1].
Table S2: The detailed results of the MEDUSA analyses.
Table S3: Diversification rates calculated using BAMM and the method-of-moments (MoM) estimators.

Figure S1. MEDUSA and BAMM results for tree 1.
Figure S2. MEDUSA and BAMM results for tree 2.
Figure S3. MEDUSA and BAMM results for tree 3.
Figure S4. MEDUSA and BAMM results for tree 4.
Figure S5. Method-of-moments estimator of net diversification rates using four different epsilon values.

Table S1: Taxa included in our analysis, based on the apoid phylogeny of Sann *et al.* [1]. The designated sampling fractions are based on the number of described species for bees (discoverlife.org [3]) and apoid wasps (Pulawski [2]). The first listed family names for the apoid wasps in the 'Family' column reflect the classification of Pulawski [2] and the second follows Sann *et al.* [1]. Quotes indicate paraphyletic groups.

specified clade	family	# described species	sample fraction (BAMM)	specified clade	family	# described species	sample fraction (BAMM)
Bees (Anthophila)				*Stizoides*	"Crabronidae" / Bembicidae	30	0.0333333333
Andreninae	Andrenidae	1553	0.000643915	*Stizus*	"Crabronidae" / Bembicidae	108	0.0092592593
Panurginae + Oxaeinae	Andrenidae	1434	0.0006973501	Crabronina + Anacrabronina	"Crabronidae" / Crabronidae	1498	0.0006675567
Anthophorini	Apidae	742	0.0013477089	Crabroninae partim	"Crabronidae" / Crabronidae	2787	0.0003588088
Apinae + Xylocopinae	Apidae	3536	0.0002828054	Dinetinae	"Crabronidae" / Crabronidae	12	0.0833333333
'Cleptoclade'	Apidae	1562	0.0006402049	Oxybelini	"Crabronidae" / Crabronidae	436	0.002293578
Colletidae	Colletidae	2667	0.0003749531	Mellininae	"Crabronidae" / Mellinidae	19	0.0526315789
Halictinae	Halictidae	3454	0.0002895194	*Diodontus*	"Crabronidae" / Pemphredonidae	76	0.0131578947
Nomiinae	Halictidae	621	0.001610306	*Passaloecus*	"Crabronidae" / Pemphredonidae	40	0.025
Nomioidinae	Halictidae	94	0.0106382979	*Pemphredon*	"Crabronidae" / Pemphredonidae	45	0.0222222222
Rophitinae	Halictidae	261	0.0038314176	*Polemistus*	"Crabronidae" / Pemphredonidae	37	0.027027027
Anthidiini	Megachilidae	883	0.0011325028	Spilomenina	"Crabronidae" / Pemphredonidae	194	0.0051546392
Dioxyini	Megachilidae	36	0.0277777778	Stigmina	"Crabronidae" / Pemphredonidae	122	0.0081967213
Lithurgini	Megachilidae	62	0.0161290323	Aphilanthopini	"Crabronidae" / Philanthidae	13	0.0769230769
Megachilini	Megachilidae	2002	0.0004995005	*Cerceris*	"Crabronidae" / Philanthidae	866	0.0011547344
Osmiini	Megachilidae	1100	0.0009090909	*Eucerceris*	"Crabronidae" / Philanthidae	41	0.0243902439
Dasypodainae	Melittidae	92	0.0108695652	Philanthini	"Crabronidae" / Philanthidae	171	0.0058479532
Melittinae + Meganomiinae	Melittidae	111	0.009009009	Pseudoscoliini	"Crabronidae" / Philanthidae	49	0.0204081633
Stenotritidae	Stenotritidae	21	0.0476190476	Odontosphecini	"Crabronidae" / Psenidae	5	0.2
				Psenini	"Crabronidae" / Psenidae	466	0.0021459227
Apoid wasps				Nyssonini + Heterogynaidae	"Heterogynaidae / Bembicidae"	236	0.0042372881
Ampulicidae	Ampulicidae / Ampulicidae	202	0.004950495	Ammophilinae	Sphecidae / Sphecidae	341	0.0029325513
Ammoplanina	"Crabronidae" / Ammoplanidae	123	0.008130081	Chloriontinae	Sphecidae / Sphecidae	20	0.05
Astatinae	"Crabronidae" / Astatidae	161	0.0062111801	Podiini	Sphecidae / Sphecidae	64	0.015625
Alyssontini	"Crabronidae" / Bembicidae	64	0.015625	Prionychini + *Isodontia*	Sphecidae / Sphecidae	141	0.0070921986
Bembecinus	"Crabronidae" / Bembicidae	195	0.0051282051	Sceliphrini	Sphecidae / Sphecidae	82	0.012195122
Bembicina partim	"Crabronidae" / Bembicidae	108	0.0092592593	*Sphex*	Sphecidae / Sphecidae	130	0.0076923077
Bembix	"Crabronidae" / Bembicidae	330	0.003030303	Stangeellinae	Sphecidae / Sphecidae	1	1
Gorytina	"Crabronidae" / Bembicidae	375	0.0026666667				
Heliocausini	"Crabronidae" / Bembicidae	8	0.125				
Spheciina 1	"Crabronidae" / Bembicidae	21	0.0476190476				
Spheciina 2	"Crabronidae" / Bembicidae	31	0.0322580645				
Stictiellina	"Crabronidae" / Bembicidae	64	0.015625				

Table S2: The detailed results of the MEDUSA analyses; *r* = net diversification rate and ε = relative extinction fraction. The tree numbers denote the four chronograms of Sann *et al.* [1].

tree #	shift #	node / clade	Ln likelihoods	*r*	ε	*r* (low)	*r* (high)	ε (low)	ε (high)
1	0	Background	-369.1555	0.0268207	0.938519	0.0233585	0.0307879	0.9159659	1
	1	All bees excluding Melittidae	-174.4469	0.0548643	0.973008	0.0476429	0.0635182	0.9569510	1
	2	*Cerceris*	-7.763307	0.1261820	n/a	0.0985749	0.1795947	n/a	n/a
	3	Crabroninae	-32.11505	0.0695645	n/a	0.0604477	0.0827852	n/a	n/a
	4	Stangeellinae	0	0	n/a	0	0.0220556	n/a	n/a
	5	*Bembix*	-6.797576	0.1281510	n/a	0.0955074	0.1913843	n/a	n/a
	6	Halictinae	-9.147143	0.1894170	n/a	0.1549725	0.2559747	n/a	n/a
2	0	Background	-368.0129	0.0279806	0.931528	0.0244718	0.0319986	0.9063898	1
	1	All bees excluding Melittidae	-188.2573	0.0431545	0.989307	0.0365118	0.0512164	0.9831566	1
	2	*Cerceris*	-7.763307	0.1272140	n/a	0.0993814	0.1810635	n/a	n/a
	3	Crabroninae	-32.08365	0.0727760	n/a	0.0632345	0.0866067	n/a	n/a
	4	Stangeellinae	0	0	n/a	0	0.0229345	n/a	n/a
	5	*Bembix*	-6.797576	0.1219320	n/a	0.0908723	0.1820962	n/a	n/a
3	0	Background	-16.33606	0.0281814	n/a	0.0220324	0.0380153	n/a	n/a
	1	All bees excluding Melittidae	-187.8051	0.0436803	0.991107	0.0366226	0.0522793	0.9859948	1
	2	*Cerceris*	-7.763307	0.1256150	n/a	0.0981320	0.1787881	n/a	n/a
	3	Stangeellinae	0	0	n/a	0	0.0218376	n/a	n/a
	4	All Apoidea except Ampulicidae and Astatidae	-384.7689	0.0379915	0.872498	0.0345182	0.0419345	0.8265501	1
	5	*Bembix*	-6.797576	0.1217770	n/a	0.0907576	0.1818662	n/a	n/a
4	0	Background	-297.9525	0.0278259	0.911233	0.0241603	0.0320606	0.8744833	1
	1	All bees excluding Melittidae	-188.1805	0.0432571	0.990052	0.0364586	0.0514831	0.9843169	1
	2	*Cerceris*	-7.763307	0.1272900	n/a	0.0994407	0.1811715	n/a	n/a
	3	Crabroninae	-31.8978	0.0702481	n/a	0.0610459	0.0836191	n/a	n/a
	4	Bembicinae except Alyssontini, Nyssonini, Heliocausini, Heterogynaidae, and Spheciina 2	-75.25951	0.0439887	0.927948	0.0342626	0.0567259	0.8654618	1
	5	Stangeellinae	0	0	n/a	0	0.0216466	n/a	n/a

Table S3: Diversification rates calculated using BAMM and the method-of-moments (MoM) estimators, from tree 3. The higher classification follows Sann *et al.* [1].

specified clade / higher classification	**BAMM results**: net diversification rate *r*, speciation rate, extinction rate, & epsilon (ε)				**MoM stem-based net diversification rate *r*** under four relative extinction fractions (ε)				**MoM crown-based net diversification rate *r*** under four relative extinction fractions (ε)			
	net div.	speciation	extinction	ε	ε = 0	ε = 0.1	ε = 0.5	ε = 0.9	ε = 0	ε = 0.1	ε = 0.5	ε = 0.9
Apidae	0.07981	0.09614	0.01632	0.17	0.09316	0.09203	0.08572	0.06844	0.09547	0.09535	0.09203	0.07562
Megachilidae	0.08167	0.09898	0.01732	0.17	0.08932	0.08819	0.08187	0.06461	0.11231	0.11216	0.10808	0.08787
Colletidae + Stenotritidae	0.08035	0.097	0.01665	0.17	0.09325	0.09201	0.08507	0.0661	0.10153	0.10139	0.09748	0.07817
Halictidae	0.08159	0.09774	0.01615	0.17	0.09915	0.09791	0.09097	0.07198	0.11405	0.1139	0.10979	0.08949
Andrenidae	0.08013	0.0963	0.01616	0.17	0.0867	0.08555	0.07919	0.06178	0.10753	0.10738	0.1033	0.08314
Melittidae	0.05083	0.05534	0.00451	0.08	0.04842	0.04746	0.04215	0.02783	0.05554	0.05542	0.05214	0.0361
Ammoplanidae	n/a	n/a	n/a	n/a	0.03848	0.03764	0.03300	0.02063	n/a	n/a	n/a	n/a
Psenidae	0.04642	0.04981	0.0034	0.07	0.04588	0.04509	0.04073	0.02886	0.05088	0.05079	0.04822	0.03559
Pemphredonidae	0.04748	0.05056	0.00308	0.06	0.05066	0.04981	0.04505	0.03211	0.05277	0.05267	0.05005	0.03714
Philanthidae	0.0474	0.05051	0.00311	0.06	0.05712	0.05627	0.05151	0.0385	0.06796	0.06785	0.06489	0.05026
Sphecidae	0.04731	0.05063	0.00332	0.07	0.05065	0.04985	0.04538	0.03322	0.05595	0.05985	0.05707	0.04337
Mellinidae	n/a	n/a	n/a	n/a	0.0224	0.02165	0.01752	0.00783	n/a	n/a	n/a	n/a
Crabronidae	0.0462	0.04957	0.00336	0.07	0.05538	0.05469	0.05084	0.04032	0.05709	0.05701	0.05498	0.0449
Bembicidae partim	0.04713	0.05042	0.00329	0.07	0.04875	0.04804	0.04406	0.0332	0.05276	0.05268	0.05043	0.03934
Nyssonini + Alyssontini + Heterogynaidae	0.04625	0.04933	0.00308	0.06	0.03702	0.0363	0.03235	0.02167	0.04282	0.04273	0.04027	0.02825
Astatidae	n/a	n/a	n/a	n/a	0.02825	0.02767	0.02443	0.01575	n/a	n/a	n/a	n/a
Ampulicidae	n/a	n/a	n/a	n/a	0.02748	0.02694	0.02392	0.01579	n/a	n/a	n/a	n/a

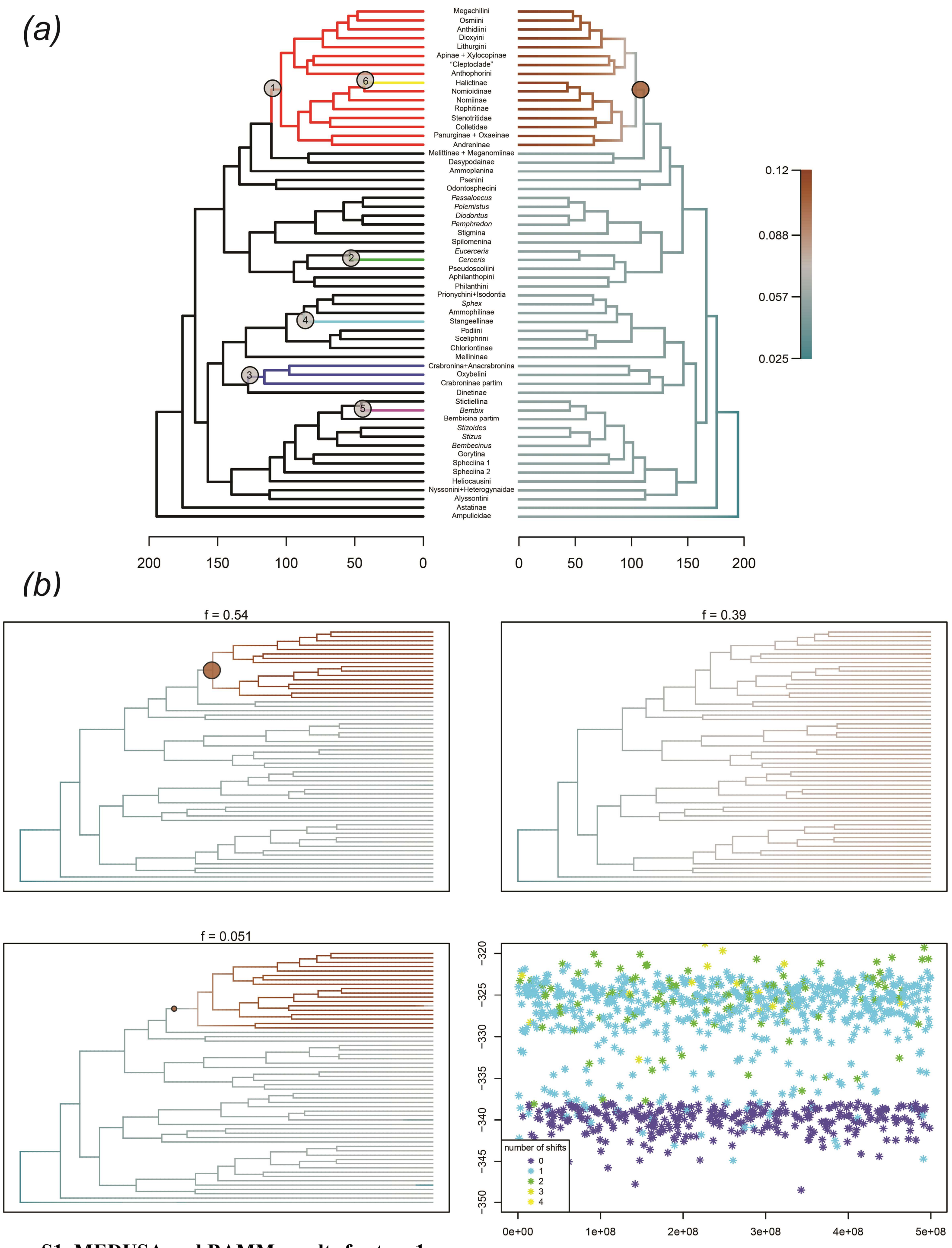

Figure S1. MEDUSA and BAMM results for tree 1.

(a) Left: MEDUSA rate shifts with black branches representing the background rate. Circles at nodes indicate up or down shifts, with values in table S3. The first rate shift is found at the base of all bees excluding Melittidae. Right: Best BAMM rate shift regime. Red colors indicate faster net diversification rates.

(b) BAMM results showing the three most probable rate shift configurations, including the best regime (top left). Circles indicate branches along which the diversification rate shifts from the background rate. The frequency of the rate shifts are indicated by "f". These configurations make up the 95% credible set of shifts, considering only core rate shifts (threshold = 5) under a prior probability of one expected rate shift. The best configuration is found at a frequency of 0.54. This regime has only one rate shift, which is found at the base of all bees excluding Melittidae. The bottom right plot shows the MCMC output, colored by the number of shifts in each sampled regime.

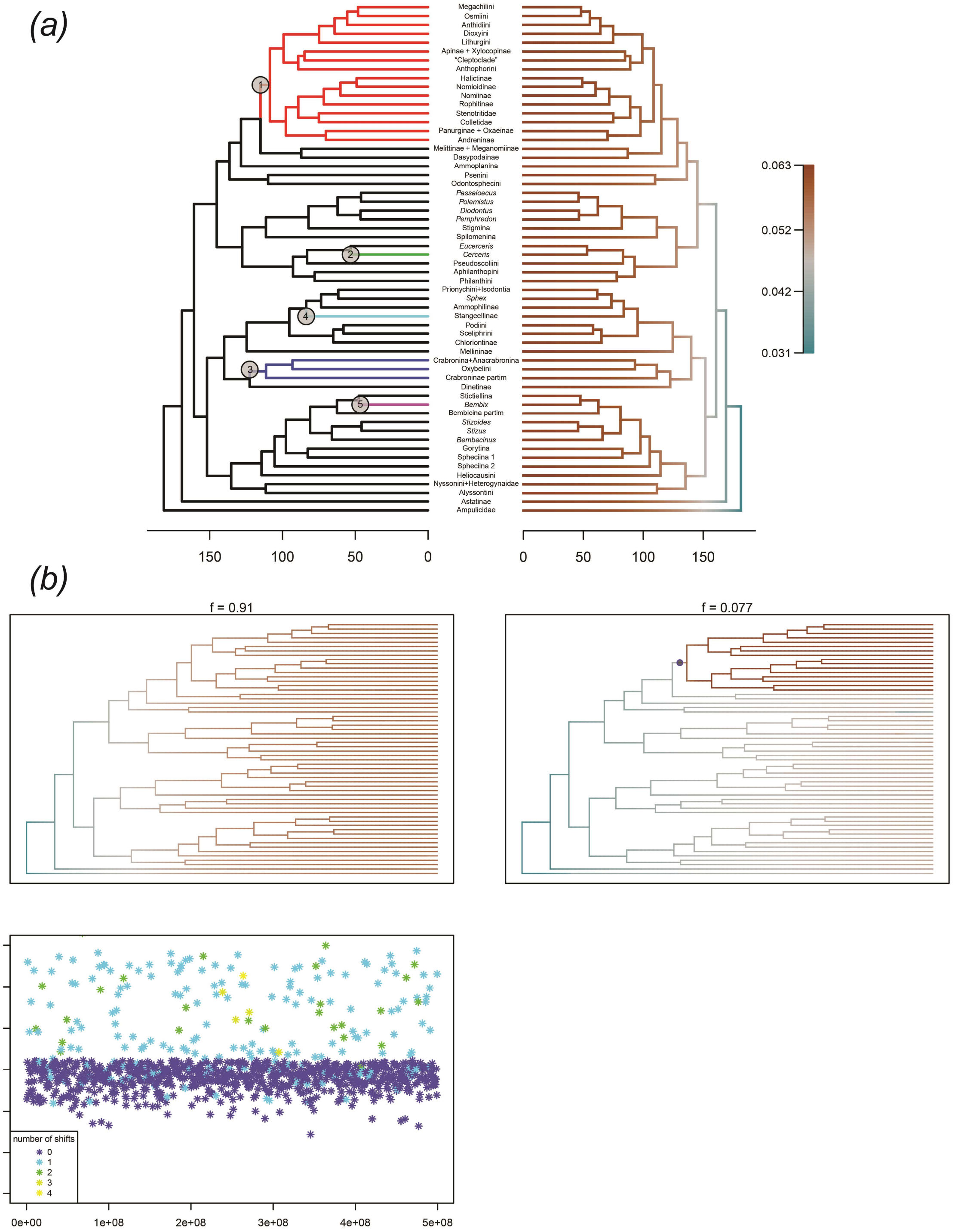

Figure S2. MEDUSA and BAMM results for tree 2.

(a) Left: MEDUSA rate shifts with black branches representing the background rate. Circles at nodes indicate up or down shifts, with values in table S3. The first rate shift is found at the base of all bees excluding Melittidae. Right: Best BAMM rate shift regime. Red colors indicate faster net diversification rates.

(b) BAMM results showing the two most probable rate shift configurations, including the best regime (top left). Circles indicate branches along which the diversification rate shifts from the background rate. The frequency of the rate shifts are indicated by "f". These configurations make up the 95% credible set of shifts, considering only core rate shifts (threshold = 5) under a prior probability of one expected rate shift. The best configuration is found at a frequency of 0.91. The best regime has no rate shift, though when there is a rate shift, it is found at the base of all bees excluding Melittidae. The bottom plot shows the MCMC output, colored by the number of shifts in each sampled regime.

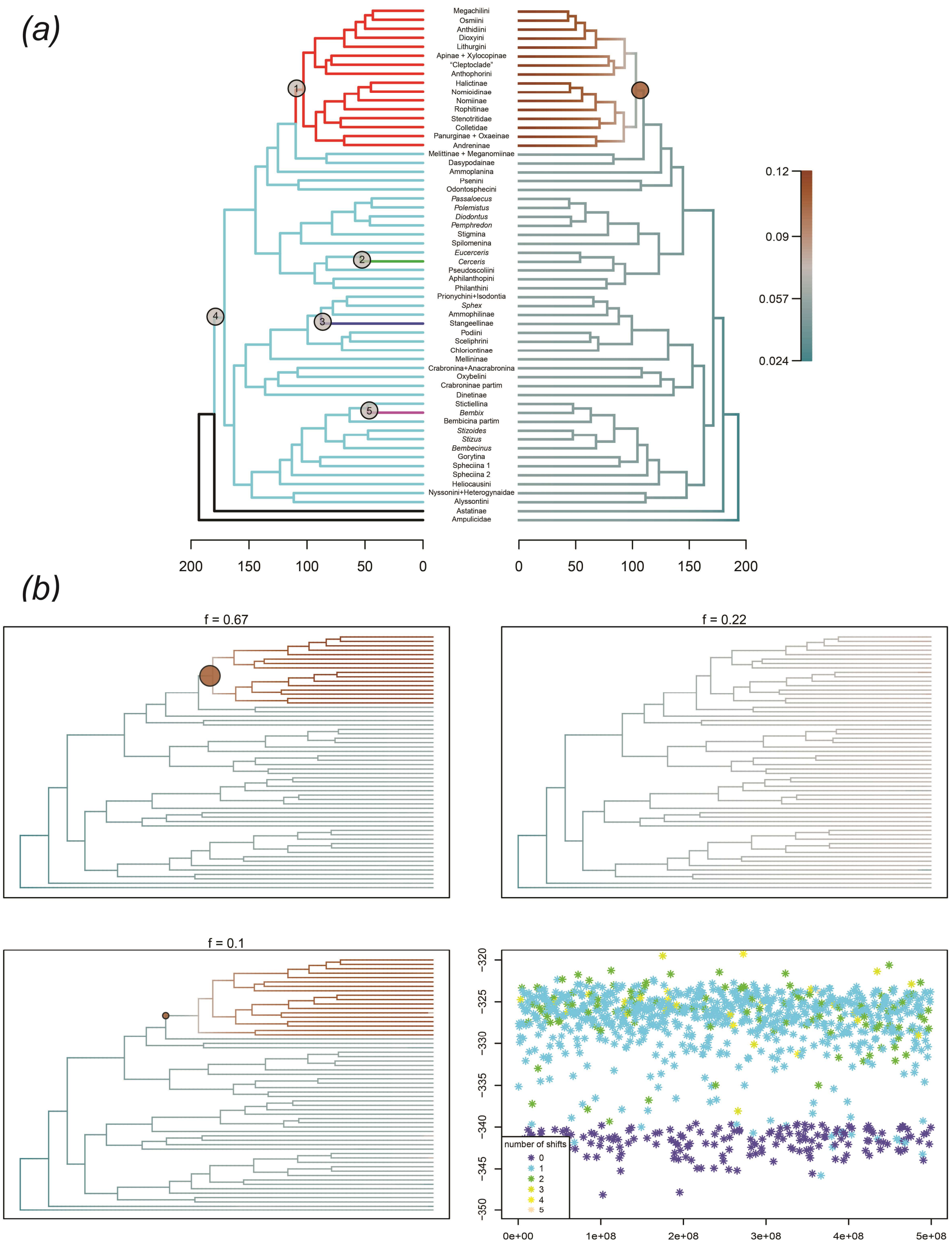

Figure S3. MEDUSA and BAMM results for tree 3.
(a) Left: MEDUSA rate shifts with black branches representing the background rate. Circles at nodes indicate up or down shifts, with values in table S3. The first rate shift is found at the base of all bees excluding Melittidae. Right: Best BAMM rate shift regime. Red colors indicate faster net diversification rates.
(b) BAMM results showing the three most probable rate shift configurations, including the best regime (top left). Circles indicate branches along which the diversification rate shifts from the background rate. The frequency of the rate shifts are indicated by "f". These configurations make up the 95% credible set of shifts, considering only core rate shifts (threshold = 5) under a prior probability of one expected rate shift. The best configuration is found at a frequency of 0.67. This regime has only one rate shift, which is found at the base of all bees excluding Melittidae. The bottom right plot shows the MCMC output, colored by the number of shifts in each sampled regime.

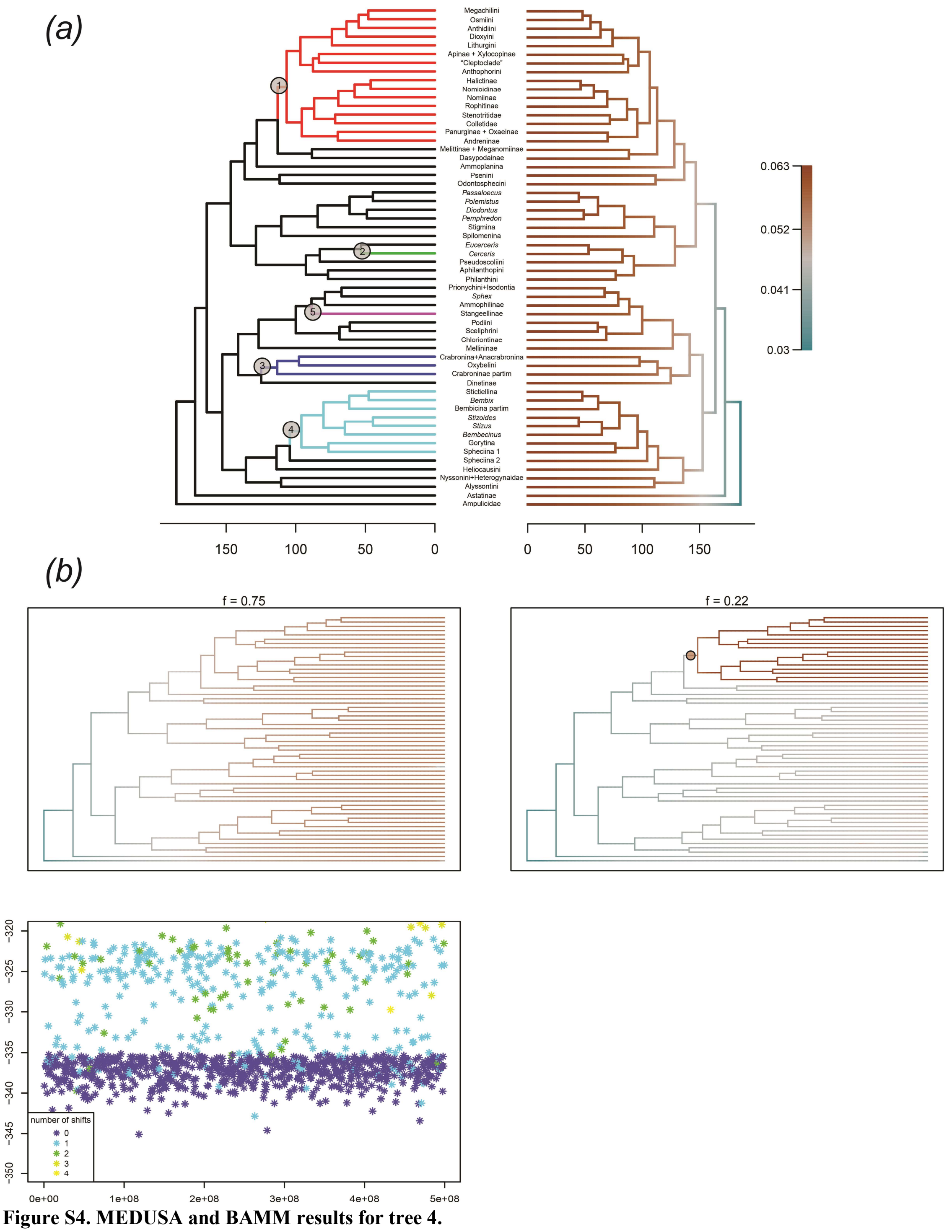

Figure S4. MEDUSA and BAMM results for tree 4.

(a) Left: MEDUSA rate shifts with black branches representing the background rate. Circles at nodes indicate up or down shifts, with values in table S3. The first rate shift is found at the base of all bees excluding Melittidae. Right: Best BAMM rate shift regime. Red colors indicate faster net diversification rates.

(b) BAMM results showing the two most probable rate shift configurations, including the best regime (top left). Circles indicate branches along which the diversification rate shifts from the background rate. The frequency of the rate shifts are indicated by “f”. These configurations make up the 95% credible set of shifts, considering only core rate shifts (threshold = 5) under a prior probability of one expected rate shift. The best configuration is found at a frequency of 0.75. The best regime has no rate shift, though when there is a rate shift, it is found at the base of all bees excluding Melittidae. The bottom plot shows the MCMC output, colored by the number of shifts in each sampled regime.

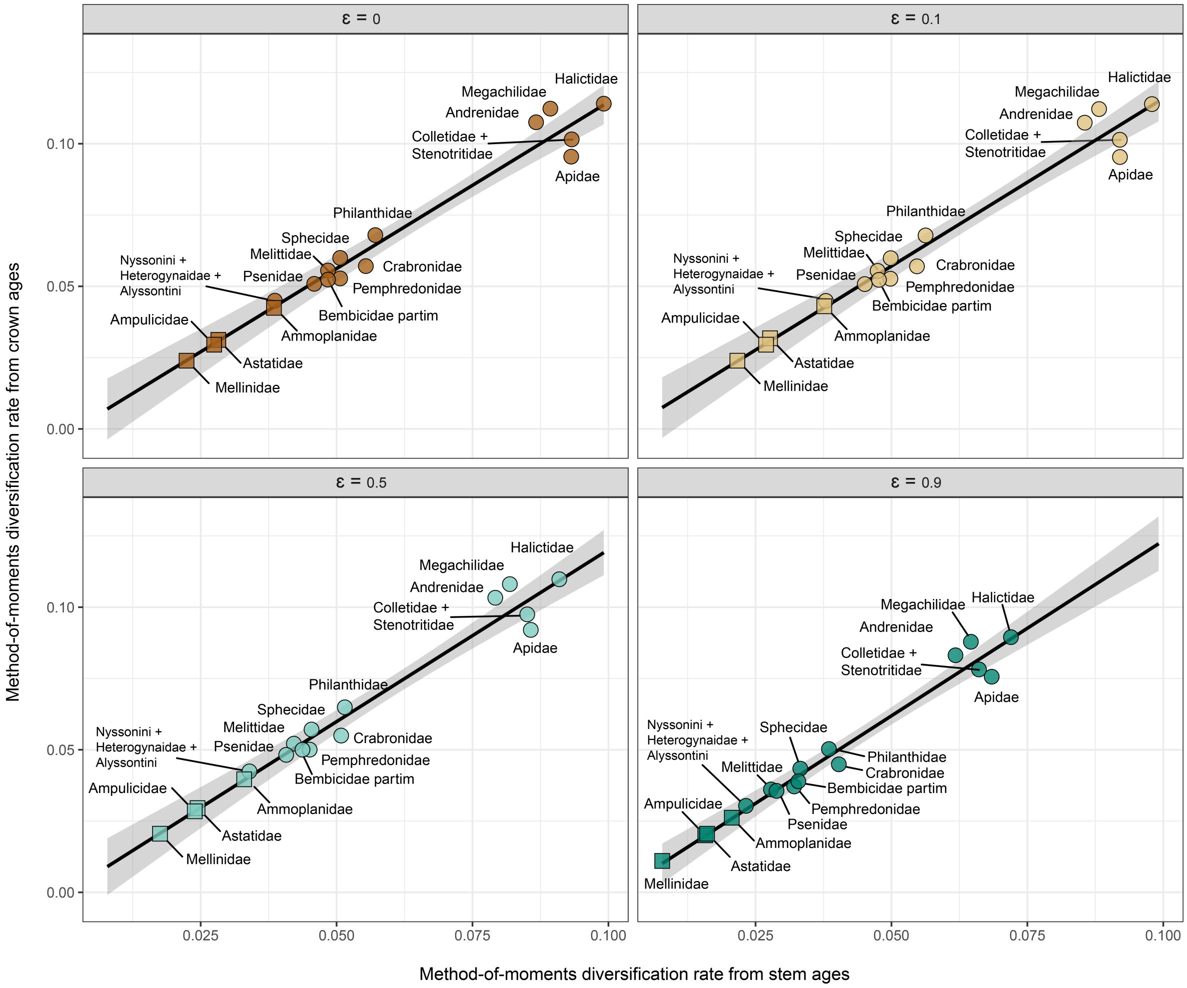

Figure S5. Method-of-moments estimator of net diversification rates using four different epsilon values. Stem-based rates are shown on the x-axis and crown-based rates on the y-axis. Four of the 17 groups did not have crown ages and are represented by square symbols. These are plotted at their stem-based rate value, but were placed directly on the regression line as an estimate of their crown-based rates. The results using an epsilon of 0.5 are also shown in figure 1c. The bee family Melittidae clusters with the apoid wasp families in respect to the stem and crown-based rates.

Supplementary references

1. Sann M *et al.* 2018 Phylogenomic analysis of Apoidea sheds new light on the sister group of bees. *BMC Evol. Biol.* **18**, 71. (doi:10.1186/s12862-018-1155-8).

2. Pulawski WJ. 2018 Catalog of Sphecidae *sensu lato*. https://www.calacademy.org/scientists/projects/catalog-of-sphecidae.

3. Ascher JS, Pickering J. 2018 Discover Life bee species guide and world checklist (Hymenoptera: Apoidea: Anthophila). http://www.discoverlife.org/mp/20q?guide=Apoidea_species.

4. Cardinal S, Danforth BN. 2013 Bees diversified in the age of eudicots. *Proc. R. Soc. B* **280**, 1-9. (doi:10.1098/rspb.2012.2686).

5. Cardinal S, Straka J, Danforth BN. 2010 Comprehensive phylogeny of apid bees reveals the evolutionary origins and antiquity of cleptoparasitism. *Proc. Natl Acad. Sci. USA* **107**, 16207-16211. (doi:10.1073/pnas.1006299107).

6. Litman JR, Praz CJ, Danforth BN, Griswold TL, Cardinal S. 2013 Origins, evolution, and diversification of cleptoparasitic lineages in long-tongued bees. *Evolution* **67**, 2982-2998. (doi:10.1111/evo.12161).

7. Branstetter MG, Danforth BN, Pitts JP, Faircloth BC, Ward PS, Buffington ML, Gates MW, Kula RR, Brady SG. 2017 Phylogenomic insights into the evolution of stinging wasps and the origins of ants and bees. *Curr. Biol.* **27**, 1019-1025. (doi:10.1016/j.cub.2017.03.027).

8. Rabosky DL. 2014 Automatic detection of key innovations, rate shifts, and diversity-dependence on phylogenetic trees. *PLoS ONE* **9**, e89543. (doi:10.1371/journal.pone.0089543.g001).

9. Rabosky DL, Grundler M, Anderson C, Title P, Shi JJ, Brown JW, Huang H, Larson JG. 2014 BAMMtools: an R package for the analysis of evolutionary dynamics on phylogenetic trees. *Methods Ecol. Evol.* **5**, 701-707. (doi:10.1111/2041-210x.12199).

10. Plummer M, Best N, Cowles K, Vines K. 2006 CODA: convergence diagnosis and output analysis for MCMC. *R News* **6**, 7-11.

11. Harmon LJ, Weir JT, Brock CD, Glor RE, Challenger W. 2008 GEIGER: investigating evolutionary radiations. *Bioinformatics* **24**, 129-131. (doi:10.1093/bioinformatics/btm538).

12. Alfaro ME, Santini F, Brock C, Alamillo H, Dornburg A, Rabosky DL, Carnevale G, Harmon LJ. 2009 Nine exceptional radiations plus high turnover explain species diversity in jawed vertebrates. *Proc. Natl Acad. Sci. USA* **106**, 13410-13414. (doi:10.1073/pnas.0811087106).

13. Magallón S, Sanderson MJ. 2001 Absolute diversification rates in angiosperm clades. *Evolution* **55**, 1762-1780. (doi:10.1111/j.0014-3820.2001.tb00826.x).

14. Meyer ALS, Wiens JJ. 2018 Estimating diversification rates for higher taxa: BAMM can give problematic estimates of rates and rate shifts. *Evolution* **72**, 39-53. (doi:10.1111/evo.13378).

15. Wiens JJ, Lapoint RT, Whiteman NK. 2015 Herbivory increases diversification across insect clades. *Nat. Commun.* **6**, 8370. (doi:10.1038/ncomms9370).
